# Long term effects of an integrated care intervention on hospital utilization in patients with severe COPD: a single centre controlled study

**DOI:** 10.1186/s12931-015-0170-1

**Published:** 2015-02-03

**Authors:** Elena Titova, Sigurd Steinshamn, Bent Indredavik, Anne Hildur Henriksen

**Affiliations:** Department of Circulation and Medical Imaging, Norwegian University of Science and Technology, Trondheim, 7006 Norway; Department of Thoracic and Occupational Medicine, Trondheim University Hospital, Trondheim, 7006 Norway; Department of Stroke, Trondheim University Hospital, Trondheim, 7006 Norway; Department of Neuroscience, Norwegian University of Science and Technology, Trondheim, 7491 Norway

**Keywords:** COPD, Pulmonary disease, Education, Self-management, Integrated care

## Abstract

**Abstract:**

Chronic obstructive pulmonary disease (COPD) is one of the main causes of morbidity and mortality globally. In Trondheim in 2008 an integrated care model (COPD-Home) consisting of an education program, self-management plan, home visits and a call centre for patient support and communication was developed. The objective was to determine the efficacy of an intervention according to the COPD-Home model in reducing hospital utilization among patients with COPD stage III and IV (GOLD 2007) discharged after hospitalization for acute exacerbations of COPD (AECOPD).

**Methods:**

A single centre, prospective, open, controlled clinical study comparing COPD-Home integrated care (IC) with usual care (UC).

**Results:**

Ninety-one versus 81 patients mean age 73.4 ± 9.3 years (57% women) were included in the IC group (ICG) and the UC group (UCG) respectively, and after 2 years 51 and 49 patients were available for control in the respective groups. During the year prior to study start there were 71 hospital admissions (HA) in the ICG and 84 in the UCG. There was a 12.6% reduction in HA in the ICG during the first year of follow-up and a 46.5% reduction during the second year (p = 0.01) compared to an 8.3% increase during the first year and no change during the second year in the ICG. During the year prior to study start, the number of hospital days (HD) was 468 in the ICG and 479 in the UCG. In the IC group, the number of HD was reduced by 48.3% during the first year (p = 0.01), and remained low during the second year of follow-up (p=0.02). In the UC group, the number of HD remained unchanged during the follow-up period. There was a trend towards a shorter survival time among patients in the ICG compared to the UCG, hazard ratio 1.33 [95% CI 0.77 to 2.33].

**Conclusion:**

Intervention according to the COPD-Home model reduced hospital utilization in patients with COPD III and IV with a persisting effect throughout the 2 years of follow-up. However, there was a trend towards a shorter survival time in the intervention group.

## Introduction

The burden of chronic obstructive pulmonary disease (COPD) is increasing, and globally COPD is predicted to become one of the main causes of morbidity and mortality in 2020 [1]. Acute exacerbations of COPD (AECOPD) are major drivers for the worsening of health status in COPD, and are important causes of hospital admissions (HA) and death [2]. Hospital admissions due to AECOPD have a negative impact on health-related quality of life (HRQOL) [3], are related to a greater decline in lung function and exercise capacity [4] and a worsening of the COPD prognosis [5,6]. Moreover, AECOPD are associated with an increased number of visits to the general practitioners (GP) or to the emergency department, and account for the greatest proportion of the total COPD burden on the health-care system. There is a direct relationship between severity of COPD and the cost of care [1]. Improving patients’ and physicians’ understanding of the nature of AECOPD, as well as the benefits of early treatment, has been shown to improve the outcomes of therapy [7].

Disease management programmes have shown promising improvements in HRQOL and reduction in hospitalisation rates and duration of hospital stays due to AECOPD [8]. However, it remains unclear which specific components of the various interventions that have the adequate power to induce a positive change, and what are the characteristics of the patients who can benefit from these interventions [9-11]. In Norway, local authorities provide free of charge home-care nursing services, aiming to provide the necessary help required for patients with chronic diseases to maintain their HRQOL by enabling them to stay in their homes for as long as desired. The most frail and severely ill patients, such as patients with severe COPD, are often users of these services. The community-based medical services may represent a suitable basis for developing and establishing an integrated care service for patients with severe COPD. The Trondheim municipality and the Department of Thoracic Medicine (DTM) at the Trondheim University Hospital (TUH) have created a collaborative project to develop and implement a model that integrates hospital- and municipal-based services for COPD patients based on local resources. The model was named the COPD-Home integrated care model [12]. In the present paper, the impact of the COPD-Home integrated care model on hospital utilization after the two-year follow-up is presented.

## Methods

### Study design

The study was a prospective, open, single-centre intervention study.

### Participants

All participants were recruited from the DTM or the Observation Unit (OU) at the TUH. The inclusion criteria were: [1] admission due to AECOPD, [2] COPD (GOLD stage III or IV, 2007), [3] living in the Trondheim municipality, [4] an ability to communicate in Norwegian, and [5] an ability to sign the informed consent form. The exclusion criterion was: [1] any serious diseases that might cause a very short lifespan (expected survival time less than six months).

All eligible patients, who gave their consent to participate in the study, were consecutively enrolled before discharge from the TUH. They were randomly allocated to either integrated care (IC) or usual care (UC) based on their address of permanent residence. The components of the COPD-Home integrated care model and strategies regarding implementation of the associated interventions have previously been described in detail [11].

### Allocation

The health-care services of Trondheim municipality are organized into four districts. The population composition of these four districts was fairly equal in age and sickness panorama (i.e. the proportion with registered multi-sickness or chronic diseases). In order to create two pairs of districts with approximately equal numbers of citizens, a pair-wise matching of districts was carried out, District Pair 1 and District Pair 2. The number of citizens aged 55–75 years was 15,800 out of 83,000 in District Pair 1 and 15,200 out of 75,000 in District Pair 2, respectively. It was decided by lottery that participants from District Pair 1 were assigned to the UC group, and participants from District Pair 2 were assigned to the IC group.

Trondheim University Hospital (TUH) is the local hospital for every person living in Trondheim. Nearly all (98-100%) patients admitted to the DTM because of AECOPD are referred as acute, not elective patients, submitted by their GP, the GP on duty or by phoning the emergency number.

The study protocol contained instructions concerning information given to the patient at discharge and did not include instructions regarding the decision to admit or discharge patients. The study nurses contacted all patients hospitalized at the DTM or the OU because of AECOPD and invited them to participate in the study provided their home address was in the municipality of Trondheim.

### Intervention

All patients hospitalized with AECOPD were treated according to the local and international guidelines. Usual care was offered to the participants allocated to the UC group and represented the standard procedure at discharge from the TUH including an evaluation by the treating physician of the patient's needs with respect to an increase in the level of care from the home-care services, rehabilitation or training or follow-up at the outpatient clinic. Moreover, the patient was given written information concerning the disease status and medication. The patient’s GP received a discharge summary with a copy to the district home-care health service if the patient was receiving any such services. The GPs followed up the COPD patients and referred the patients to a pulmonary specialist if necessary.

Home-care nursing services include help with personal care, medicine dosage, injections, specific disease monitoring and observation, assistance with meals, help in physical training and help in coordinating aid efforts with other services and emergency assistance, as well as supervision regarding the using of security alarms. The participants in the UC group were evaluated by a study coordinator (a specialist nurse) at discharge from TUH and during scheduled visits in their own homes after six-, 12- and 24 month of follow-up. The specialist nurses are registered nurses with enhanced competence in respiratory medicine.

Integrated care intervention in accordance with the COPD-Home model was offered to the participants allocated to the IC group. The core elements of the COPD-Home model were: [1] a call centre staffed by three specialist nurses for support and communication with patients and home-care nurses, and coordination between the various levels of care. The patients were routinely (at least once a month) contacted by the specialist nurses, and they were supported by telephone calls during COPD exacerbations; [2] an education session for home-care nurses: a three-hour theoretical session covering several aspects of COPD and two days of practice at the DTM; [3] an interactive 15-minute e-learning program for the patients concerning the management of COPD; [4] an individualized self-management plan introduced to the patient at discharge by the treating doctor and a specialist nurse. The plan contains tools for the monitoring of symptoms and written instructions for the self-initiation of prednisone and/or antibiotics, provided specific symptoms have been recorded; and [5] joint visits at the patients home by the specialist nurse (together with the home-care nurse for participants receiving home services) at approximately three days, 14 days, six months, 12 months and 24 months post-discharge. The major components of these visits were repetition of the core elements of the education program, making necessary changes in the patient’s treatment plans and the reinforcing of specific health behaviors. The patient’s GP was also invited to participate in these visits.

All participants included in the COPD-Home study were free to use all available medical services, including their GPs.

### Outcome measures

The primary outcomes were: [1] Number of hospital admissions caused by AECOPD (HA) and [2] number of in-hospital days (HD) due to AECOPD. HA and HD due to AECOPD include admittances where pneumonia was diagnosed during the hospital stay. The HA and HD was assessed in three time periods: during the one year prior to the study enrolment, and during the first- and second year of follow-up. Data concerning HA and HD were collected from the hospital registry database’s medical charts. The International Classification of Disease (ICD 10) was used to classify the diseases that caused the hospitalization. A HA was defined as a hospitalization due to any acute worsening of COPD, including pneumonia (ICD-10: J44.09 or J13-18 + J44.0-9).

Data from patients who completed a minimum of two years of follow-up were included in the analysis. The medical records from the one-year period prior to the study start and the index hospitalizations were reviewed to identify the Charlson’s co morbidities by using the ICD-10 coding algorithm [13-15]. Conditions that occurred or were diagnosed during the follow-up period were not included. The age-adjusted Charlson Comorbidity Index (CCI) was calculated by use of an online calculator (farmacologiaclinica.info/scoring-tools/).

Baseline characteristics (age, gender, forced expiratory volume in one second (FEV1), arterial blood gases, the body mass index (BMI) and baseline data on co-morbidity, medical treatment, lifestyle factors, as well as patient characteristics and disease status after one and two years of follow-up, were collected from the study reports. Data regarding mortality were collected from medical records at the TUH and the National Cause of Death Registry, Statistics Norway.

### Ethics

The study was approved by the Regional Ethics Committee (REC), and the participants gave their written informed consent. Mortality was not a primary outcome of the study, but when the patients’ data were analysed after two years of follow-up, an increased number of deaths were registered among the patients in the IC group compared to the UC group. The REC was informed, and the study was s temporarily stopped. Data on the causes of death were analysed, and the REC concluded that the increased number of deaths in the IC group was not related to the COPD-Home intervention, but could be explained by pre-study poorer health status and higher age. The study was reopened after a break of eight months, and the project manager decided not to continue the intervention program, but continued the registration of observations in both groups until all the participants had been followed- up for three years.

### Statistical analysis

Demographic characteristics registered at both baseline and follow-up were reported as mean ± SD. Data with a normal distribution were compared using an independent sample *t*-test while categorical variables were analysed using the chi-square test.

The percentage of the differences in the number of HA and HD was calculated by dividing the absolute difference between the values from the year prior to the study start and the values after one or two years of follow-up in the IC and UC groups respectively.

Due to the skewed distribution, HA and HD were summarized using medians and inter-quartile ranges (IQR). Comparisons of HA and HD between the UC and IC groups were performed using non-parametric techniques.

Patients were categorized according to the frequency of their HA, using HA category 1 (≤1 HA per year) and HA category 2 (≥2 HA per year). The differences in the proportions of individuals in the HA categories during the first and second year of follow-up were tested using the chi-squared test, and the results were presented as percentages with corresponding p-values.

A univariate survival analysis for the different demographic and clinical variables at the study start was performed according to the Kaplan Meier procedure, whereas tests for the differences in the survival curves for the different categories of variables were performed with Mantel-Cox statistics. Variables with a significant effect on survival in the univariate analysis were further analysed using the multivariate Cox proportional hazard regression model to calculate the hazard ratios and 95% confidence intervals for risk factors. Survival analyses were performed with total mortality (all causes of death) as the endpoint. P-values < 0.05 were considered to be statistically significant, and all statistical analyses were performed using PASW Statistics 19, SPSS Inc. Chicago.

## Results

**Figure 1:**
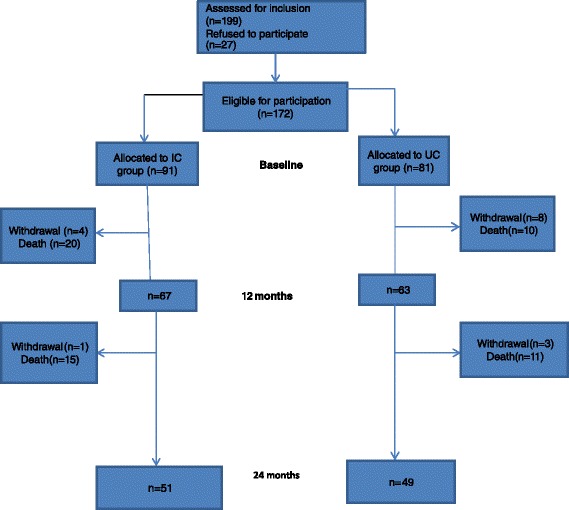
**Schematic presentation of the study profile over two years.** IC: integrated care; UC: usual care.

Between March 2008 and November 2011, a total of 199 patients were invited to participate in the study. Of those, 27 patients declined to participate, and 172 patients were randomly allocated to the IC group (n = 91) or the UC group (n = 81), respectively. After two years of follow-up, a total of 100 patients (58% of the included patients), 51 patients in the IC group and 49 patients in the UC group, were available for evaluation (Figure 1).

There were no significant differences in baseline characteristics between the IC and the UC groups, but when comparing those who died during follow-up with those who were still alive after two years, those who died had a significantly higher PaCO2 and lower BMI at baseline (Table 1). Those who died during follow-up also had significantly more HA and HD during the year prior to study start.

**Table 1:** **Baseline characteristics of participants in the integrated and usual care groups according to follow-up status**

	**Died during follow-up**			**Alive after two years of follow-up**			
	**IC group**	**UC group**	**p-value**	**IC group**	**UC group**	**p-value**	**p - value**
	**n = 35**	**n = 21**	**IC v UC**	**n = 51**	**n = 49**	**IC v UC**	**Died v Alive**
Age, years, mean ± SD	76.4 ± 9.6	74.7 ± 8.0	0.5	72.5 ± 9.0	73.1 ± 9.4	0.7	0.09
Sex: female, n (%)	20 (57)	13 (62)	0.7	29 (57)	27(55)	0.8	1.0
Living arrangements:							
Living alone, n (%)	15 (42.9)	12 (57.1)	0.3	29 (56.9)	22 (44.9)	0.2	0.7
Receiving home-care services, n (%)	24 (58.5)	17 (41.5)	0.3	24 (48.0)	20 (40.8)	0.5	0.5
FEV1, litres, mean ± SD	0.81 ± 0.4	0.75 ± 0.3	0.5	0.92 ± 0.35	0.84 ± 0.34	0.3	0.07
FEV1%, mean ± SD	32.3 ± 10.5	31.7 ± 9.9	0.9	34.9 ± 9.4	33.4 ± 9.2	0.4	0.2
PaO_2_, kPa, mean ± SD	8.04 ± 1.3	8.7 ± 1.7	0.1	9.0 ± 1.4	8.7 ± 1.2	0.3	0.3
PaCO _2_,kPa, mean ± SD	6.1 ± 1.3	6.2 ± 1.3	0.9	5.6 ± 0.8	5.7 ± 1.0	0.1	0.008
GOLD stage 4, n (%)	14 (40)	11 (52)	0.4	20 (39)	22 (45)	0.6	0.5
BMI, kg/m^2^, mean ± SD	21.6 ± 4.7	22.1 ± 6.5	0.8	23.9 ± 5.4	23.9 ± 4.6	0.9	0.05
Current smoker, n (%)	10 (28.6)	11 (52.4)	0.08	18 (35.3)	15 (30.6)	0.8	0.6
Inhaled medication:							
LAMA	13 (37.1)	11 (52.4)	0.3	20 (39.2)	25 (51)	0.2	0.8
LABA + ICS	25 (71.4)	17 (81.0)	0.4	36 (70.6)	35 (71.4)	0.9	0.6
HA one year prior to study start, median (IQR)	2 (1, 3)	2 (1, 3)	0.6	1.0 (1, 1)	1.0 (1, 2)	0.03	0.01
HD one year prior to study start, median (IQR)	9 (6, 22)	11 (7.5, 18.5)	0.9	7 (4, 11)	8 (5, 13)	0.2	0.003

IC: integrated care; UC: usual care; FEV_1_: forced expiratory volume in one second; FEV1%: FEV1% of predicted value; PaCO _2_: partial pressure of carbon dioxide in the arterial blood; PaO _2_: partial pressure of oxygen in the arterial blood; BMI: body mass index; LAMA: long- acting muscarinic receptor antagonist; LABA: long-acting β_2_- agonist; ICG: Inhaled Corticosteroids; AECOPD: acute exacerbation of COPD; HA: hospital admissions due to AECOPD; HD: hospital days due to AECOPD.

During the year prior to study start, there were 71 HA at the DTM in the IC group and 84 in the UC group. When comparing the number of HA at the DTM during follow-up with the year prior to study start, there was a 12.6% reduction in the IC group during the first year of follow-up and a 46.5% reduction during the second year (p = 0.01). In the UC group there, was an 8.3% increase in HA during the first year and no change in the number of HA during the second year of follow-up (Table 2). During the year prior to study start, the number of HD was 468 in the IC group and 479 in the UC group (Table 2). In the IC group, the number of HD was reduced by 48.3% during the first year (p = 0.01), and remained low during the second year of follow-up (p = 0.02). In the UC group, the number of HD remained unchanged during the follow-up period. During the year prior to study start, the percentages of patients in HA category 1 (≤1 HA per year) and HA category 2 (≥2 HA per year) were similar in both the IC and the UC group. During the first year of follow-up, eight (15.7%) patients changed from HA category 2 to HA category 1 in the IC group, while two patients (4%) changed from HA category 1 to HA category 2 in the UC group, p = 0.01 (Table 3). During the second year of follow-up, there was still a trend towards a higher percentage of patients in HA category 1 in the IC group compared to the UC group (Table 3).

**Table 2:** **Changes in hospital utilisation during the two years follow-up period**

	**One year before study start**	**First year of follow-up**	**Differences (%)**	p – value*	**Second year of follow-up**	**Differences (%)**	p - value**
HA (N), IC group	71	62	- 12.6	0.13	38	- 46.5	0.01
HA (N), UC group	84	91	+8,3	0.61	84	0	0.33
HD (N), IC group	468	242	- 48.3	0.01	244	- 47.8	0.02
HD (N), UC group	479	488	+1.8	0.19	466	- 2.7	0.20

P -value*: the year before study start versus the first year of follow-up.

p -value**: the year before study start versus the second year of follow-up.

IC: integrated care; UC: usual care; HA: hospital admissions due to AECOPD; HD: hospital days due AECOPD.

**Table 3:** **Changes in the proportions of individuals in the two hospital admission categories during follow-up**

	**IC group (n=51)**		**UC group (n=49)**		**p-value**
**HA category**	**1**	**2**	**1**	**2**	
The year prior to study start, n, (%)	40 (78.4)	11 (21.6)	39 (79.6)	10 (20.4)	1.00
The first year of follow-up, n (%)	48 (94.1)	3 (5.9)	37 (75.5)	12 (24.5)	0.01
The second year of follow-up, n (%)	44 (86.3)	7 (13.7)	37 (75.5)	12 (25.4)	0.21

HA: hospital admissions due to AECOPD; HA category 1: ≤ 1 HA per year;

HA category 2: ≥ 2 HA per year.

There were no significant differences in the number of current cigarette smokers between the IC group and the UC group at baseline or within the groups during the follow-up period, but there was a trend towards a reduction in the number of current smokers in the IC group from 18 (35.5%) at baseline to 16 (31.4%) after 12 months and to 14 (27.5%) after 24 months. In the UC group the corresponding numbers were 15 (30.6%) at baseline, unchanged after 12 months and 13 (26.5%) after 24 months.

There was a non-significant increase in the number of patients receiving long-acting muscarinic receptor antagonist (LAMA) during 24 months of follow-up in the IC group from 20 (39.2%) to 25 (49.05%) and a trend towards a reduction in the UC group from 25 (51.0%) to 23 (46.9%). At baseline 36 (70.6%) in IC group versus 35 (71.4%) in UC group received a combination of long-acting β_2_-agonist (LABA) combined with inhaled corticosteroids (ICS), and there were no significant differences either between the IC and the UC group or within the groups during the follow-up period.

During the two-year follow-up period, there was a trend towards an increased number of deaths in the IC group compared to the UC group, 35 (38.5%) and 21 (25.9%), respectively. In both groups, the most frequent primary cause of death was AECOPD and/or pneumonia, 22 patients in the IC group and 10 patients in the UC group respectively (Table 4).

**Table 4:** **All-cause mortality during two years of follow-up**

**Causes of death/group**	**IC group**	**UC group**	P-value*
	**(n=35)**	**(n=21)**	
AECOPD and pneumonia, n (%)	22(62.8)	10(47.6)	0.26
Cardiovascular diseases, n (%)	4(11.4)	4(19)	0.43
Sepsis, not related to infections in lungs, n (%)	4(11.4)	2(9.5)	0.82
Cancer, n (%)	3(8.6)	1(4.8)	0.59
Mors subita, nonviolent, causa ignota, n (%)	2¤(5.7)	4¤(19)	0.12

¤Death certificates are not made public; *statistically significance IC group versus UC group.

IC: integrated care; UC: usual care.

The univariate survival analysis for the different demographic and clinical variables showed that survival time was significantly related to baseline age (p = 0.04), BMI (p = 0.006), PaCO _2_ (p = 0.02) and receiving home-care nursing (p = 0.001). The shortest survival time was found in patients receiving home-care nursing (p = 0.001), with a high age (≥80 years), a low BMI and a high PaCO_2_ (Table 5). The longest survival time was found in patients 70–79 years old, with a PaCO _2_ < 6.1 and a BMI 18–25. In multivariate survival analysis, mortality was significantly related to a lower BMI (p = 0.008) and receiving home-care nursing (p = 0.017), Table 6.

**Table 5:** **Univariate survival analysis for the different demographic and clinical variables**

**Long- rank test**	**Total no. **	**No. dead**	**75% percentiles of survival time (months)**	**P - value**
**Groups:**				
**IC group**	86	35	12.0	0.17
**UC group**	70	21	19.0	
**Sex:**				0.71
**Male**	67	23	17.0	
**Female**	89	33	13.0	
**Age (yrs):**				0.04
**<70**	49	14	18	
**70-79**	54	16	21	
**=>80**	53	26	9	
**BMI:**				0.006
**<18**	20	13	3.0	
**18-25**	95	31	16.0	
**>25**	41	12	23.0	
**FEV1:**				0.34
**<0,5L**	11	4	13.0	
**0,5L-1,0L**	103	41	12.0	
**>1,0**	41	11	24.0	
**FEV1%:**				0.58
**<30**	58	23	16.0	
**30 -54**	97	33	14.0	
**PaCO **_**2**_ **,** **kPa:**				0.02
**<=6.1**	108	32	18.0	
**>6.1**	47	23	11.0	
**PaO**_**2**_ **, kPa:**				0.17
**<=8.0**	53	23	12.0	
**>8.0**	103	33	17.0	
**Civil status:**				0.72
**Living alone**	78	16	16.0	
**Living with partner**	78	12	12.0	
**Home-care nursing:**				0.001
**No**	71	15	9.0	
**Yes**	85	41		
**Charlson co morbidity index:**				0.23
**1 - 3**	32	9	13.0	
**4 - 6**	94	33	17.0	
**7 - 10**	30	14	8.0	

IC: integrated care; UC: usual care; BMI: body mass index; FEV1: forced expiratory volume in one second; FEV1%: FEV1% of predicted value; PaCO _2_: partial pressure of carbon dioxide in the arterial blood; PaO _2_: partial pressure of oxygen in the arterial blood.

**Table 6:** **Multivariate survival analysis for the different demographic and clinical variables**

**Variable**	**Group**		**Sex**		**Age(yrs)**			**BMI**			**PaCO**_**2**_ **, kPa**		**Home-care nursing**	
	**UC group**	**IC group**	**Male**	**Female**	**<70**	**70-79**	**≥80**	**<18**	**18-25**	**>25**	**≤6.1**	**>6.1**	**No**	**Yes**
**Unadjusted hazard ratios (univariate) Total (95% CI)**	1	1.46 (0.85-2.51)	1	1.10 (0.65-1.88)	0.52 (0.27-0.99)	0.52 (0.28-0.96)	1	2.49 (1.30-4.78)	1	0.85 (0.44-1.66)	1	1.87 (1.09-3.19)	1	2.80 (1.55-5.07)
**Adjusted (multivariate) hazard ratio Total (95% CI)**	1	1.33 (0.77-2.33)	1	0.75 (0.43-1.32)	0.80 (0.40-1.66)	0.57 (0.30-1.13)	1	3.09 (1.55-6.17)	1	0.96 (0.47-1.99)	1	1.81 (1.01-3.24)	1	2.10 (1.10-4.04)

IC: integrated care; UC: usual care; BMI: body mass index; PaCO _2_: partial pressure of carbon dioxide in the arterial blood.

Although the difference was not statistically significant, there was a trend towards a shorter survival time among the participants in the IC group compared to those in the UC group with a hazard ratio of 1.33 [95% CI 0.77 to 2.33].

At the department of thoracic medicine (DTM) at TUH the extra costs attached to the COPD-Home intervention amounted to roughly one 100% nurse position (workdays from 8 am to 3 pm) during four years including training of the home care nurses. During the inclusion period the Municipality of Trondheim spent a total of ca 40.000 € in extra hours for home care nurses attending training programs at the DTM.

## Discussion

The aim of the present study was to evaluate the long-term impact of the integrated care intervention according to the COPD-Home model on hospital utilization in patients with severe and very severe COPD. The core elements of the COPD-Home model were disease-specific education, an individualized self-management plan, joint visits in the patient’s home by a specialist nurse, and a call centre support. This intervention model includes at least four of the six necessary key components of the ATS chronic care model [16]. In addition, the COPD-Home model contains dimensions specifically targeted at multi-morbid COPD patients, such as coordinating services between different health-care levels and health-care providers, including nursing homes.

The principal findings of the present study were a reduction in the number of patients with frequent HA and a reduced number of both HA and HD during follow-up in the IC group compared to the UC group. While the decrease in the number of HD was more pronounced during the first year of follow-up, the decrease in the number of HA became significant only during the second year of follow-up. In contrast, in the UC group there was no significant changes in the number HA or HD during the follow-up period.

When comparing our results with results from previous studies on integrated care interventions among COPD patients, we found that the interpretation is hampered by differences in inclusion criteria, the scope of the various interventions and the duration of the follow-up period. In a study by Bourbeau and colleagues from 2003 [17], the intervention program included a self-management plan supported by an experienced case manager and communication by regular telephone contact. In the intervention group, they found an improvement of the patient’s health status, 39.8% reduction in hospital admissions for AECOPD and 57.1% reduction in hospital admissions for other health problems. While in the present study only patients with a very short life expectancy were excluded, Bourbeau and colleagues did not include patients with a history of congestive heart failure or asthma as well as patients who had recently attended a respiratory rehabilitation program or patients in long-term care facilities. Moreover, in contrast to the present study where patients were included during a hospital stay because of an AECOPD, in the study by Bourbeau only patients with a stable COPD for at least four weeks were enrolled.

Casas and colleagues recruited exacerbated COPD patients at discharge from hospital to a standardized integrated care intervention. They found a significantly lower re-hospitalization rate during the 12 months of follow-up in the intervention group compared to the control group, 1.5 ± 2.6 versus 2.1 ± 3.1 respectively [18]. In contrast to the present study, patients with severe co morbidities were excluded from the study by Casas. However, the overall percentages of deaths during the 12 months of follow-up were rather similar, 19% versus 16% in the intervention and control groups respectively. Moreover, in 2010, Rice and colleagues reported the results from an intervention study in which patients with severe COPD received one education session, an action plan for self-treatment and monthly telephone calls from a case manager [19]. After one year of follow-up, they found a 41% reduction in the hospitalization rate and the number of emergency visits for COPD among the patients in the intervention group. The positive effect of their disease management intervention is very similar to the results achieved in the present study, but the number of deaths during the one-year follow-up period was relatively lower and more evenly distributed between the intervention group (9.7%) and the control group (12.9%). The differences in mortality between the two studies may be explained by a higher age and the more severe disease status at inclusion among the participants in the present study compared to the study by Rice and colleagues.

More recently, Fan and colleagues evaluated the efficacy of a comprehensive care management program in reducing the risk for COPD related hospitalizations [20]. The authors were unable to show any reduction in hospital admissions, and surprisingly the all causes mortality was significantly higher among the patients in the intervention group compared to those in the control group, HR: 3.00 (95% CI 46–6.17). The authors did not find a satisfactory explanation for the increase in HR, but deaths due to COPD accounted for the largest difference between the two groups. Also, it was revealed that patients in the intervention group did not take medication any sooner in the event of an AECOPD than the patients in the usual care group. As in the present study, there were rather few exclusion criteria, but in the study by Fan and colleagues the patients’ education program was more extensive. Moreover, the qualifications of the case managers ranged from nurse practitioners to medical doctors while in the present study only specialist nurses acted as case managers.

When interpreting the results of the COPD-Home intervention, we found that hospital admissions were reduced only in a proportion of the patients.

In COPD-Home highly qualified specialist nurses were available for support, guidance and education at a low threshold basis, for both patients and home care nurses. These essential parts of the intervention may have encouraged behavioral changes among the patients. Bourbeau and colleges have addressed the importance of achieving behavioral changes among the patients as a prerequisite to succeed in improving the health outcomes [21,22]. Also, the specialist nurses’ easy access to discuss their actions with a pulmonologist may have been an important factor in providing a more customized treatment for the patients. Hence, we speculate that the reduction in hospital utilization in the integrated care group was a result of better knowledge, skills and confidence to manage their own healthcare. However, due to the restricted number of participants it is beyond the scope of the present study to conclude what particular parts of the intervention that led to the reduced hospital utilization.

Information concerning the number and duration of the COPD exacerbations, as well as the time from onset of symptoms until the start of self-initiated treatment is insufficient due to the many incomplete registrations in “My COPD book”. Therefore, it cannot be concluded why the intervention was successful in some patients and ineffective in others. However, one hypothesis is that some patients had knowledge and confidence beforehand, and when given the intervention with education and supervision they became prepared to play a more active role in managing their own health resulting in improved disease control [23]. On the other hand, it is likely that patients who were not capable of, or motivated for such changes did not benefit from an intervention programs relaying on active involvement from the patients themselves [24]. Also, it might be difficult for patients with several co morbidities and patients of advanced age to distinguish the origin of their various symptoms, such as shortness of breath or pain [25,26].

The COPD-Home intervention was ambitious, and some measures were not successful. The GPs did not find time to meet with the specialist nurses in the patients homes after discharge, and a substantial number of persons were involved in the home-care services for each patient because of the continuous change of work hours among the home care nurses. As a result not all the involved home care nurses managed to attend the COPD-Home education program. We therefore assume that the qualifications and skills of the home-care nurses varied substantially resulting in differences in their guidance of the patients with respect to adherence to their action plans [23].

COPD is a multi-component disease in its nature. Also, COPD patients with co-morbidities such as cancer or cardiovascular disorders may require a specific integrated pathway of health care across several medical specialties [27]. More knowledge is needed concerning what it takes for particularly multi-morbid, older patients to become informed, motivated, and involved in their own healthcare. In future studies, these aspects need to be taken into account in order to construct more facilitated intervention programs that contain schemes for the registration of complex symptoms and alternative treatment strategies [24].

## Conclusions

An intervention in accordance with the COPD-Home integrated care model among patients with severe COPD was shown to be effective in reducing the number of patients with frequent hospital admissions and the number of hospital admissions as well as in-hospital days. Hence, the intervention contributed to a reduction in hospital utilization during the two-year follow-up period. Even so, we may not be able to claim generalizability of the results because of the relatively small number of participants with a considerable variety of co-morbidities and health-care needs. Additionally, the finding of a trend towards increased mortality in the intervention group that could not be fully explained by pre-study health differences further emphasizes that our results must be interpreted with caution.

The study highlights the necessity for careful individually designed self-management programs. In future research, subsets of COPD patients that are most receptive to such interventions need to be identified, and the effectiveness of intervention programs targeting complex co-morbid patients should be studied.

## Abbreviations

COPD: Chronic obstructive pulmonary disease
HA: Hospital admission
IQR: Inter-quartile ranges
IC: Integrated care
UC: Usual care
AECOPD: Acute exacerbation of chronic obstructive pulmonary disease
HRQoL: Health-related quality of life
DTM: Department of Thoracic Medicine
TUH: Trondheim University Hospital
GP: General practitioner
HD: in-hospital days
FEV1: Forced expiratory volume in one second
FEV1%: Forced expiratory volume in one second % predicted
REC: Regional Ethics Committee
CCI: Charlson Comorbidity Index
PaCO _2_: Partial pressure of carbon dioxide in the arterial blood
PaO _2_: Partial pressure of oxygen in the arterial blood
BMI: Body mass index
LABA: Long-action β_2_-agonist
LAMA: Long-acting muscarinic receptor antagonist
ICS: Inhaled Corticosteroids
HR: Hazard ratio
95% CI: 95% confidence interval
